# Direct-Acting Antiviral Drugs Reduce Fibromyalgia Symptoms in Patients with Chronic Hepatitis C

**DOI:** 10.3390/jcm11185327

**Published:** 2022-09-10

**Authors:** Kuo-Tung Tang, Ching-Chun Lin, Yi-Hsing Chen, Tsai-Ling Liao, Der-Yuan Chen, Sheng-Shun Yang, Chia-Chang Chen

**Affiliations:** 1 Division of Allergy, Immunology, and Rheumatology, Taichung Veterans General Hospital, Taichung 407, Taiwan; 2 Faculty of Medicine, National Yang Ming Chiao Tung University, Taipei 112, Taiwan; 3 Ph.D. Program in Translational Medicine and Rong Hsing Research Center for Translational Medicine, College of Life Sciences, National Chung Hsing University, Taichung 402, Taiwan; 4 Department of Family Medicine, China Medical University Hospital, Taichung 404, Taiwan; 5 Department of Family Medicine, School of Medicine, College of Medicine, China Medical University, Taichung 404, Taiwan; 6 Department of Post-Baccalaureate Medicine, College of Medicine, National Chung Hsing University, Taichung 402, Taiwan; 7 Department of Medical Research, Taichung Veterans General Hospital, Taichung 407, Taiwan; 8 Rheumatology and Immunology Center, China Medical University Hospital, Taichung 404, Taiwan; 9 College of Medicine, China Medical University, Taichung 404, Taiwan; 10 Institute of Medicine, Chung Shan Medical University, Taichung 402, Taiwan; 11 Division of Gastroenterology and Hepatology, Department of Internal Medicine, Taichung Veterans General Hospital, Taichung 407, Taiwan

**Keywords:** direct-acting antiviral drugs, fibromyalgia, hepatitis C, pain

## Abstract

Background Fibromyalgia (FM) is a complex disorder characterized by chronic widespread pain and significant patient burden. Patients with chronic hepatitis C are reportedly predisposed to the development of FM. Direct-acting antiviral drugs (DAA) achieved a remarkable therapeutic efficacy in CHC patients. We therefore investigated the impact of DAA on FM symptoms in CHC patients. Methods We enrolled consecutive CHC patients who received DAA. FM symptoms were evaluated based on the 2016 American College of Rheumatology (ACR) fibromyalgia scale at baseline and 12 and 24 weeks after cessation of DAA therapy. Logistic regression was performed to determine the influence of HCV on FM at baseline. We also recruited individuals who underwent a health checkup examination as the control group, and calculated the standardized prevalence ratio of FM in CHC patients. Comparisons of fibromyalgia in different time points were undertaken using the Wilcoxon signed-rank test. Results A total of 33 CHC patients (15 males and 18 females) and 402 controls were recruited. All CHC patients achieved sustained virological response. Two (6%) patients and two (0.5%) controls fulfilled the diagnostic criteria for FM, and the standardized prevalence ratio was 23.9 in CHC patients. Logistic regression also showed increased odds for FM in CHC patients after adjusting for age and sex (OR: 14.4; 95%CI: 1.6, 128.0). In addition, their fibromyalgianess scale decreased at 12 and 24 weeks after DAA therapy. In conclusion, CHC patients were more likely to develop FM. Implementation of DAA therapy might improve FM symptoms in these patients.

## 1. Introduction

Fibromyalgia (FM) is a complex disorder characterized by chronic widespread pain and accompanying sleep problems, fatigue and cognitive dysfunction [1]. Epidemiological data showed that the prevalence of FM in the general population is 1–2% and leads to impaired quality of life in affected patients [1]. To date, the disease pathogenesis is not fully elucidated and treatment is far from satisfactory [2]. Dysfunctional processing of pain in the brain has been speculated to be involved in the pathogenesis of FM. In addition, previous studies revealed increased circulating levels of pro-inflammatory cytokines, such as interleukin-1R antibody (IL-1Ra), IL-6 and IL-8, in FM patients [3,4]. IL-6 could induce hyperalgesia, fatigue and depression, while IL-8 promotes sympathetic pain. Therefore, the dysregulation of these cytokines may play a role in the generation of FM symptoms.

Several rheumatic and infectious diseases have concomitant FM [5,6]. Studies have found an increased prevalence of FM in patients with chronic hepatitis C (CHC) when compared with healthy controls [7,8,9,10]. The prevalence of FM was reported to be 8–19% in CHC patients. These findings might be explained by the production of pro-inflammatory cytokines in these patients. Indeed, previous studies revealed increased levels of pro-inflammatory cytokines, including IL-6 and IL-8, in patients with CHC [11,12]. Furthermore, chronic infection per se has been postulated to be associated with a maladaptive behavior pattern, which in turn causes anxiety, sleep disturbance and physical deconditioning. These factors may also predispose individuals to the development of FM [13]. Nevertheless, there exists the knowledge gap with respect to the pathogenic mechanism of FM symptoms in CHC patients.

CHC is endemic in Taiwan [14,15]. However, the prevalence of FM in Taiwanese CHC patients has not been investigated. Recent emergence of the direct-acting antiviral drugs (DAA) has revolutionized the management of CHC [16,17]. DAA are associated with a remarkable virologic response, which in turn leads to a reduction in liver-related morbidities, the incidence of hepatocellular carcinoma, the need for liver transplantation and mortality. CHC is associated with a variety of extrahepatic manifestations, including cryoglobulinemia and arthritis, among others [18]. Previous studies have demonstrated an improvement of extrahepatic manifestations after treatment with DAA in CHC patients [19]. Universal reimbursement for DAA in CHC patients has been implemented in the national health insurance system of Taiwan since June 2019. We hypothesized that FM is more prevalent in Taiwanese CHC patients when compared with the general population and that treatment with DAA could improve FM symptoms in these patients. Therefore, we aim to investigate concomitant FM in Taiwanese CHC patients who received DAA.

## 2. Materials and Methods

### 2.1. Study Participants

We recruited consecutive CHC patients before they received DAA at our Division of Gastroenterology and Hepatology from March 2020 through January 2022. Patients with concurrent human immunodeficiency virus infection were excluded from the study. The sustained virologic response was defined as an undetectable viral level 12 weeks after the cessation of DAA therapy. We also recruited individuals who underwent a health checkup examination at our hospital from November 2020 through January 2021 to estimate the prevalence of FM in the general population. This study complied with the Declaration of Helsinki and was approved by the Institutional Review Board of Taichung Veterans General Hospital (approval No. CE20052A and CE20279B). All participants were Han Chinese and received a complete explanation of the study and provided written informed consent.

### 2.2. Liver Function Test and Hepatitis C Virus (HCV) Viral Loads

Serum levels of alanine aminotransferase (ALT) and aspartate aminotransferase (AST) were determined by spectrophotometry (Fujifilm, Osaka, Japan). The upper limit of normal range is 35 U/L for ALT and 38 U/L for AST. Serum HCV DNA was extracted using a High Pure Viral Nucleic Acid kit (Roche, Mannheim, Germany) and then quantified by Roche Cobas TaqMan HCV Test (Roche Diagnostics, Basel, Switzerland).

### 2.3. Serum Levels of Inflammatory Cytokines in CHC Patients

Serum levels of IL-6 and IL-8 were measured by the Quantikine HS and QuantiGlo enzyme-linked immunosorbent assay (ELISA) kit (R&D Systems, Minneapolis, MN, USA).

### 2.4. Evaluation of Concomitant Fibromyalgia in CHC Patients

Each subject was asked to complete a questionnaire comprising of the 2016 revision to the 2010/2011 American College of Rheumatology (ACR) criteria for fibromyalgia [20,21,22]. The questionnaire is meant to be used in epidemiological research to diagnose FM. The questionnaire is composed of two parts: the widespread pain index (WPI) and the symptom severity (SS) score. WPI delineates the extent of body pain, while SS score describes the associated symptoms, including cognitive symptoms, fatigue, waking unrefreshed, headache, pain or cramps in lower abdomen and depression. The WPI > 7 and SS score > 5 or WPI 3 to 6 and SS score > 9 fulfills for the diagnosis of FM given that pain is generalized (at least 4 of 5 regions) and present for at least 3 months. We also summed the WPI and SS score together to obtain the fibromyalgianess scale, which represents the severity of FM symptoms [21]. The fibromyalgianess scale is used to estimate the severity of FM symptoms (polysymptomatic distress) in an individual regardless of whether he/she fulfils the FM diagnostic criteria, and is well correlated with physical function and quality of life [23]. The 2011/2016 ACR FM criteria questionnaire was re-administered 12 and 24 weeks after DAA therapy in CHC patients. 

The validated Chinese version of revised Fibromyalgia Impact Questionnaire (FIQR) (Cronbach’s α: 0.95 in 103 Taiwanese FM patients; Whei-Mei Shih, personal communication) was used to evaluate relevant symptoms, functional impairment and the well-being of patients within recent week in FM patients [24]. It is comprised of 21 questions and the maximum FIQR score is 100. For CHC patients who fulfilled the diagnosis of FM based on the 2011/2016 ACR criteria, FIQR was administered at baseline and 12 weeks after cessation of DAA therapy.

### 2.5. Statistical Analysis

Numerical data were presented as mean ± standard deviation (SD) if normally distributed and median plus interquartile range (IQR) if not. Categorical data were presented as the percentage. Comparisons between groups on numerical variables were undertaken using the Student’s *t* test if normally distributed and using the Mann–Whitney U test if not. Categorical variables were compared between groups using the chi square test. The Fisher’s exact test is undertaken instead if there is at least one cell in the contingency table of the expected frequencies below 5. Comparisons of numerical variables in different time points were undertaken using the Wilcoxon signed-rank test. Logistic regression was performed to determine the influence of HCV on FM at baseline, adjusting for age and sex. The standardized prevalence ratio for FM was calculated for CHC patients based on the age and sex distributions of Taiwanese population (2021 census data) and the prevalence of FM in age- and sex-specific strata derived from either the CHC patients or the control group. All statistical analyses were performed using Stata, version 14.0 (StataCorp, College Station, TX, USA). 

## 3. Results

### 3.1. Baseline Characteristics

The study participants’ baseline characteristics are presented in Table 1. A total of 33 CHC patients (15 males and 18 females) received DAA. Most (73%) of them received sofosbuvir-velpatasvir. Their median serum ALT/AST levels were 44 (IQR: 35, 106) and 43 (IQR: 27, 77) U/L, respectively. The median HCV viral load was 3.3 × 10^6^ (IQR: 1.5 × 10^6^, 6.5 × 10^6^) IU/mL. In terms of complications, 4 (12%) had liver cirrhosis and 2 (6%) had hepatocellular carcinoma. All of them achieved sustained virologic response. The virus clearance was maintained through 24 weeks after cessation of DAA therapy in all patients. We also recruited 402 individual (185 females and 217 males) as the control group. CHC patients were older than the control group (mean age: 58 vs. 48 years). The body mass index and proportion of co-morbid rheumatic diseases was similar between these two groups. One (3%) of the CHC patients had co-morbid systemic lupus erythematosus, manifested as leukopenia and thrombocytopenia. 

### 3.2. The Prevalence of FM in CHC Patients and the Control Group at Baseline

**Figure 1:**
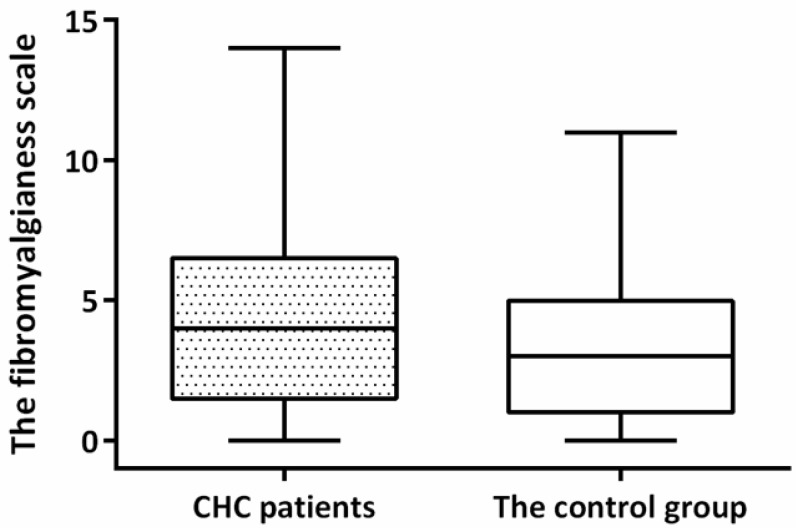
Comparison of fibromyalgianess between CHC patients at baseline and controls. CHC, chronic hepatitis C.

Two individuals fulfilled the diagnosis of FM in CHC patients and in the control group, respectively. The prevalence of FM was 6% in CHC patients, significantly higher than that (0.5%) in the control group by the Fisher’s exact test. Nonetheless, the fibromyalgianess scale was not higher in CHC patients when compared with that in the control group (Figure 1), although these patients had worse cognitive symptoms (Table S1). Logistic regression also showed an increased odds for FM in CHC patients after adjusting for age and sex (OR: 14.4; 95%CI: 1.6, 128.0). The standardized prevalence ratio for FM in CHC patients based on the prevalence of FM derived from the control group was 23.9 (95%CI: 2.9, 86). 

### 3.3. Factors Associated with FM in CHC Patients at Baseline

**Figure 2:**
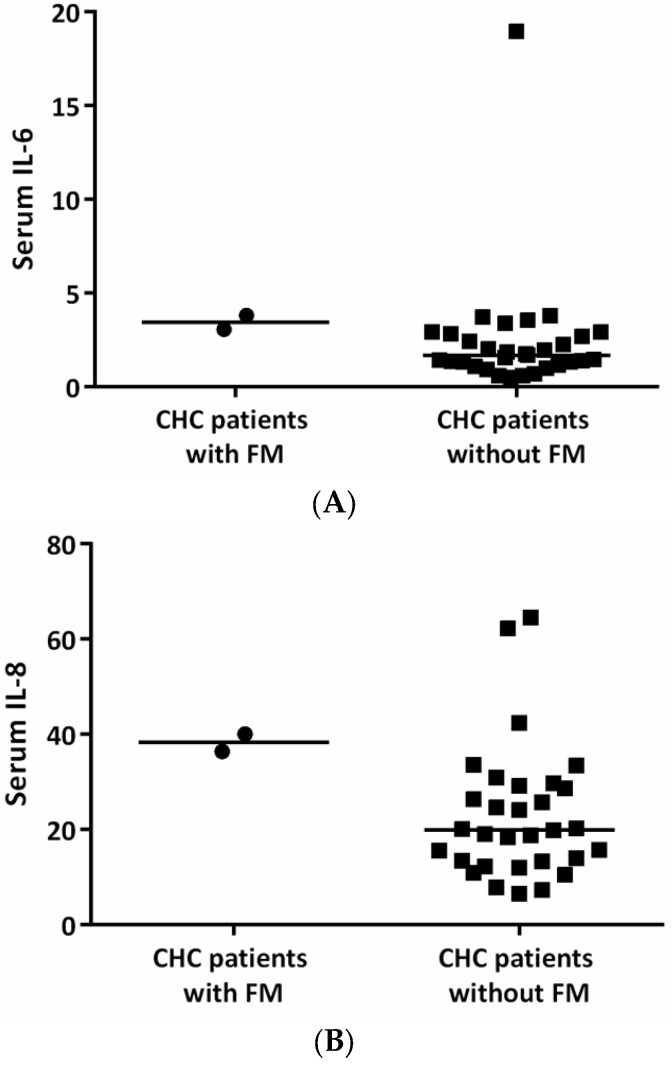
Serum levels of (**A**) IL-6 (pg/mL) and (**B**) IL-8 (pg/mL) between CHC patients with and without FM at baseline. CHC, chronic hepatitis C; FM, fibromyalgia; IL, interleukin.

As shown in Figure 2, there was a trend toward an increase in serum levels of IL-6 and IL-8 in CHC patients with FM when compared with those patients without FM (both *p* = 0.05). CHC patients with FM had higher liver function tests (serum levels of ALT and AST) when compared with those patients without FM, although statistical significance was not achieved (both *p* = 0.08). There was no difference in demographic characteristics and the base 10 logarithm of HCV viral loads between CHC patients with and without FM (Table 2). To be noted, one of the CHC patients with FM had the highest body mass index (35.1) among all CHC patients. 

### 3.4. The Impact of DAA Therapy on FM Symptoms in HCV Patients

**Figure 3:**
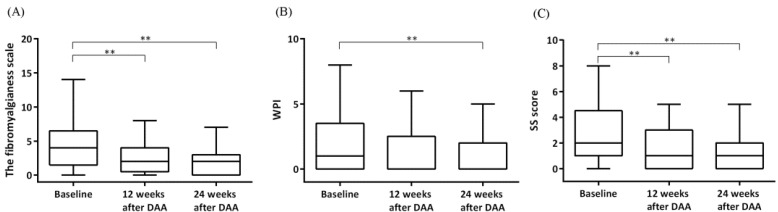
Comparison of (**A**) fibromyalgianess, (**B**) widespread pain index (WPI), and (**C**) symptom severity (SS) score between baseline, 12 and 24 weeks after cessation of DAA therapy in CHC patients. CHC, chronic hepatitis C; DAA, direct-acting antiviral drugs. ** *p* < 0.001 by the Wilcoxon signed rank test.

The fibromyalgianess scale decreased at 12 and 24 weeks after DAA therapy in CHC patients (Figure 3a). In addition, both the WPI and SS score decreased at 24 weeks after DAA therapy (Figure 3b,c). When we further analyzed subdomains of the SS score (Table 3), sleep disturbance, headache and lower abdominal pain improved at 12 and 24 weeks after DAA therapy compared with those at baseline. Fatigue improved at 24 weeks after DAA therapy when compared with that at baseline. Nevertheless, depression did not significantly improve at both 12 and 24 weeks after DAA therapy. Among two CHC patients with FM, their fibromyalgia scale decreased (17 and 14 at baseline decreased to 15 and 13, respectively, at both 12 and 24 weeks after DAA therapy). Nevertheless, they still fulfilled the FM diagnostic criteria. Their FIQR score also decreased 12 weeks after DAA therapy (30 and 15 at baseline decreased to 23 and 13 respectively).

## 4. Discussion

Chronic infection may contribute to the pathogenesis of FM. We found a higher prevalence of FM in CHC patients when compared with the control group. In addition, DAA therapy not only eradicated the virus but also improved FM symptoms in CHC patients. 

In addition to hepatic injury, HCV infection also affects other organ systems. A variety of rheumatic manifestations has been reported and includes cryoglobulinemia, arthritis, sicca, etc. [25]. Previous studies have demonstrated the benefits of CHC therapy on these extrahepatic manifestations. Earlier studies found that the eradication of HCV by interferon-based therapy could improve cryoglobulinemic vasculitis [26,27] and related arthritis [25]. Since the introduction of the first DAA in 2011, they have now become the treatment of choice in CHC patients [28,29]. Use of DAA could achieve the cure of CHC in more than 95% of the patients and such effective viral eradication could prevent the development of liver cirrhosis, hepatocellular carcinoma and mortality. Moreover, Bonacci et al. followed 46 CHC patients with cryoglobulinemic vasculitis and 42 CHC patients with asymptomatic cryoglobulinemia [19]. They observed a reduction in cryoglobulinemia and vasculitis activity 24 months after DAA therapy. Other groups also found a complete clinical response rate of 64–88% in DAA-treated patients with CHC-related cryoglobulinemia 12–24 weeks later [30,31]. In addition, tender and swollen joint counts improved in 24 patients with CHC-related arthritis after DAA therapy [32]. Furthermore, DAA therapy could even reduce the disease activity of concomitant rheumatic diseases (mostly rheumatoid arthritis) [33]. However, the impact of HCV eradication on concomitant FM has not yet been explored. Our observations showed that DAA therapy leads to a decrease in FM symptoms (fibromyalgianess) 12 and 24 weeks after its cessation. Furthermore, the two CHC patients diagnosed with concomitant FM reported improved fibromyalgianess scale and FIQR after DAA therapy, whereas fibromyalgianess scale and FIQR did not improve with time in a retrospective analysis of 20 FM patients regularly followed at our Division of Allergy, Immunology, and Rheumatology (Figure S1). This is the first report of the possible beneficial effect of DAA therapy on FM symptoms in CHC patients. Studies of a larger sample size are required before the conclusion is made. 

The beneficial effect of DAA therapy on FM symptoms among CHC patients may be mediated through several mechanisms. We found that both WPI and SS score improved after DAA therapy. Moreover, fatigue, sleep disturbance, headache and lower abdominal pain improved in these patients. Fatigue is a common symptom in CHC patients and its prevalence ranges from 52% to 71% [34,35,36,37,38]. Additionally, fatigue was reported by 37% of our CHC patients at baseline. One study demonstrated improvement of fatigue in 401 CHC patients 24 weeks after the completion of interferon-based therapy [38]. Another study reported improvement of fatigue in 105 CHC patients receiving DAA therapy [39]. Our observation is resonant with these findings. Sleep disturbance has been reported in 95% of CHC patients [40]. In our CHC patients, 42% reported waking unrefreshed at baseline, which was reduced to 27% at 24 weeks after DAA therapy. Taken together, DAA therapy is associated with a possible reduction in FM symptoms in CHC patients due to both alleviation of body pain and improvement of associated symptoms. 

Several studies have found an increased prevalence of FM in CHC patients based on the 1990 ACR diagnostic criteria, at 8–19% [7,8,9,10]. The 2011/2016 ACR criteria, which was more sensitive and better in the diagnostic performance [41], was used to diagnose FM in our study. The prevalence of FM was 6% in our CHC patients, significantly higher than that in the control group. One may argue that the control group may not well represent the general Taiwanese population. Nevertheless, the prevalence of FM (0.5%) in our control group was similar to that in the literature regarding the general population [42]. CHC is postulated to contribute to FM based on several mechanisms. Upregulation of pro-inflammatory cytokines may be one of the mechanisms and has been reported in both FM patients [43] and CHC patients [11,12]. In line with this, our observation showed a trend toward higher serum levels of IL-6 and IL-8 in CHC patients with FM than those patients without FM. A behavioral pattern which leads to mood and sleep disturbance has also been implicated. However, we did not observe a worsening of sleep problems and depression in CHC patients when compared with the control group. Instead, we found an increase in cognitive symptoms in these CHC patients. The pathophysiology of FM is characterized by brain dysfunction [44,45]. Could these cognitive symptoms be linked to the development of FM? More studies are needed. In the present study, interestingly, one of the CHC patients with FM had the highest body mass index among all CHC patients. Obesity has long been recognized as a potential risk factor for the development of FM and is prevalent in CHC patients [46]. It is possible that obesity may contribute to the concomitant FM in CHC patients.

Our study is limited by several factors. First, the sample size is small and our findings need confirmation in a larger study. In particular, the FM cases in CHC patients are too few to determine the influence of CHC on the development of FM and of DAA therapy on these FM patients. To avoid bias in the logistic regression for rare events, we also undertook penalized logistic regression [47] and similarly found increased odds for FM in CHC patients after adjusting for age and sex (OR: 12.9; 95%CI: 1.8, 91.1) (data not shown). Second, there is no longitudinal data with respect to CHC patients receiving no antiviral therapy. Nevertheless, this is unethical due to the efficacy of DAA therapy and the governmental policy of universal treatment in CHC patients. Third, the diagnosis of FM was not made by a physician. However, the 2011/2016 ACR criteria based on a self-report has good sensitivity and specificity and can be used in the epidemiological research [22].

In conclusion, our results demonstrated an increased prevalence of FM in CHC patients when compared with the control group. DAA therapy might improve FM symptoms in these CHC patients. Larger studies are required to validate our findings.

## Figures and Tables

**Table 1:** Baseline characteristics of study participants.

	CHC Patients (*n* = 33)	The Control Group (*n* = 402)
Age, mean (SD) *	58 (11)	48 (13)
Body mass index, median (IQR)	24.2 (22.5, 26.8)	23.7 (21.4, 26.2)
Female sex, n (%)	18 (55)	185 (46)
Rheumatic diseases, n (%)	1 (3)	13 (3)
Chronic hepatitis C, n (%)	33 (100)	2 (0.5)
HCV viral load, median (IQR) (IU/mL)	3.3 × 10^6^ (IQR: 1.5 × 10^6^, 6.5 × 10^6^)	N.A.
Direct-acting antiviral drugs, n (%)		
Sofosbuvir-velpatasvir	24 (73)	N.A.
Glecaprevir-pibrentasvir	6 (18)	N.A.
Elbasvir-grazoprevir	3 (9)	N.A.

CHC, chronic hepatitis C; N.A., not available. * *p* < 0.05.

**Table 2:** Baseline characteristics between CHC patients with and without fibromyalgia.

	CHC Patients with Fibromyalgia (*n* = 2)	CHC Patients without Fibromyalgia (*n* = 31)
Age	55	58 (48, 64)
Body mass index	30.5	24.2 (21.6, 26.8)
Female sex, n (%)	1 (50)	17 (55)
Serum levels of alanine aminotransferase (U/L)	172	43 (32, 97)
Serum levels of aspartate aminotransferase (U/L)	127	42 (26, 69)
HCV viral load, mean (SD) (IU/mL)	1.6 × 10^6^	3.8 × 10^6^ (1.5 × 10^6^, 7.7 × 10^6^)

CHC, chronic hepatitis C; HCV, hepatitis C virus. Numerical variables are presented as the median only for 2 CHC patients with fibromyalgia, and the median plus interquartile range for 31 CHC patients without fibromyalgia.

**Table 3:** Subdomains in the symptom severity scale at baseline and after DAA therapy in CHC patients.

Subdomains, *n* (%)	Baseline	12 Weeks after DAA Therapy	24 Weeks after DAA Therapy
Cognitive symptoms			
No	15 (45)	19 (58)	19 (58)
Mild	16 (48)	12 (36)	12 (36)
Moderate	2 (6)	2 (6)	2 (6)
Fatigue			
No	20 (61)	22 (67)	24 (73) *
Mild	9 (27)	9 (27)	8 (24)
Moderate	4 (12)	1 (3)	1 (3)
Severe	0 (0)	1 (3)	0 (0)
Waking unrefreshed			
No	19 (58)	20 (61) *	24 (73) *
Mild	6 (18)	11 (33)	7 (21)
Moderate	8 (24)	2 (6)	2 (6)
Headache	15 (45)	8 (24) *	5 (15) *
Pain or cramps in lower abdomen	8 (24)	1 (3) *	3 (9) *
Depression	5 (15)	3 (9)	2 (6)

CHC, chronic hepatitis C; DAA, direct-acting antiviral drugs. * *p* < 0.05 when compared with that at baseline, by the Wilcoxon signed rank test.

## Data Availability

The data that support the findings of this study are available upon request from the corresponding author.

## Supplementary Materials

The following supporting information can be downloaded at: https://www.mdpi.com/article/10.3390/jcm11185327/s1, Figure S1. Comparison of (a) fibromyalgianess and (b) revised Fibromyalgia Impact Questionnaire (FIQR) score with time in 20 patients with fibromyalgia (mean age: 47 ± 8 years; 19 females and 1 male). Table S1: Comparisons of subdomains in the symptom severity scale between CHC patients at baseline and the control group.
