# Supplementary material for: Direct-Acting Antiviral Drugs Reduce Fibromyalgia Symptoms in Patients with Chronic Hepatitis C
Source: J Clin Med. 2022 Sep 10;11(18):5327. doi: 10.3390/jcm11185327 (PMC9500682; doi:10.3390/jcm11185327)
Supplement: Supplementary file 1 [file jcm-11-05327-s001.zip › jcm-1895187-supplementary.pdf]

Table S1. Comparisons of subdomains in the symptom severity scale between CHC patients at baseline and the control group.

Subdomains, n (%)	CHC patients (n=33)	The control group (n=402)
Cognitive symptoms*		
No	15 (46)	281 (70)
Mild	16 (49)	119 (30)
Moderate	2 (6)	2 (1)
Fatigue		
No	20 (61)	222 (55)
Mild	9 (27)	162 (40)
Moderate	4 (12)	18 (5)
Waking unrefreshed		
No	19 (58)	197 (49)
Mild	6 (18)	182 (45)
Moderate	8 (24)	21 (5)
Severe	0 (0)	2 (1)
Headache	15 (46)	163 (41)
Pain or cramps in lower abdomen	8 (24)	85 (21)
Depression	5 (15)	38 (10)

CHC, chronic hepatitis C

*$p<0.05$ by the Mann-Whitney U test.

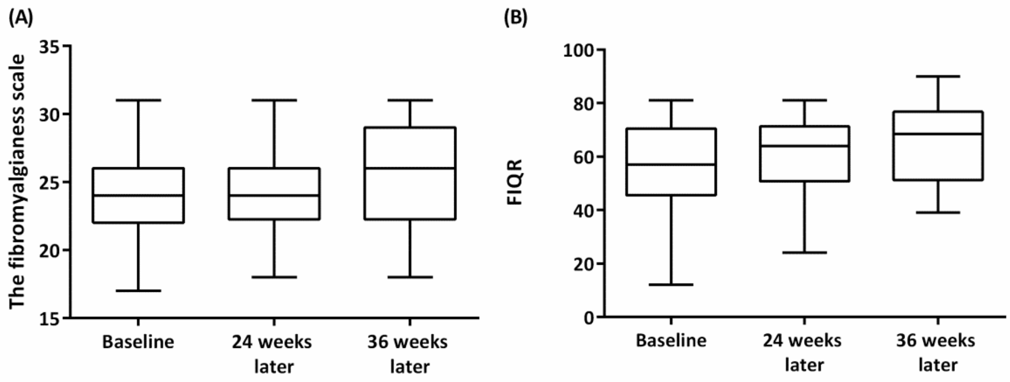

Figure S1. Comparison of (a) fibromyalgianess and (b) revised Fibromyalgia Impact Questionnaire (FIQR) score with time in 20 patients with fibromyalgia (mean age: 47 ± 8 years; 19 females and 1 male).
